# Dairy consumption and ovarian cancer risk in the Netherlands Cohort Study on Diet and Cancer

**DOI:** 10.1038/sj.bjc.6602890

**Published:** 2005-11-22

**Authors:** M Mommers, L J Schouten, R A Goldbohm, P A van den Brandt

**Affiliations:** 1 Department of Epidemiology, NUTRIM, Maastricht University, PO Box 616, Maastricht 6200 MD, The Netherlands; 2 Department of Food and Chemical Risk Analysis, TNO Quality of Life, Zeist, The Netherlands

**Keywords:** ovarian neoplasms, dairy products, milk, lactose, dairy fat

## Abstract

Ovary cancer risk in relation to consumption of dairy products was investigated using a self-administered questionnaire on dietary habits and other risk factors for cancer, which was completed in 1986 by 62 573 postmenopausal women participating in the Netherlands Cohort Study. Follow-up for cancer was implemented by annual record linkage with the Netherlands Cancer Registry and a nationwide pathology registry. After 11.3 years of follow-up, data of 252 incident epithelial ovarian cancer cases and 2216 subcohort members were available for analysis. No association was seen between consumption of milk, yoghurt, cheese or fermented dairy products and ovarian cancer risk. The multivariable adjusted relative risk of epithelial ovarian cancer for women in the highest compared to the lowest quintile of intake of lactose or dairy fat was 0.93 (95% confidence interval (CI)=0.60–1.45; *P*_trend_=0.32) and 1.53 (95% CI=1.00–2.36; *P*_trend_=0.11), respectively. Lactose or dairy fat intakes were not associated with serous ovarian cancer risk. Our results do not support an association between consumption of dairy products or lactose intake and ovarian cancer.

Ovarian cancer ranks fifth as the most common malignancy among women in Europe, with approximately 34 500 newly diagnosed ovarian cancer cases in 1998 (Ferlay *et al*, 1999). Due to its late detection in many cases, survival rates are low.

In an ecological analysis, ovarian cancer incidence and per capita milk consumption have been found to be correlated among populations (Cramer, 1989). The results from case–control and prospective cohort studies regarding milk consumption and ovarian cancer risk have been inconsistent (Mettlin and Piver, 1990; Webb *et al*, 1998; Kushi *et al*, 1999; Cramer *et al*, 2000; Goodman *et al*, 2002; Fairfield *et al*, 2004; Larsson *et al*, 2004; Qin *et al*, 2005).

As a possible mechanism underlying the effect of milk consumption on ovarian cancer risk, metabolites of the milk-sugar lactose have been suggested (Cramer, 1989; Cramer *et al*, 1989). With the exception of two case–control studies that reported a protective effect (Herrinton *et al*, 1995; Goodman *et al*, 2002), most studies found no statistically significant effect of high lactose intake on overall ovarian cancer risk (Engle *et al*, 1991; Webb *et al*, 1998; Kushi *et al*, 1999; Cramer *et al*, 2000; Cozen *et al*, 2002; Fairfield *et al*, 2004). On the other hand, for serous epithelial ovarian tumours, a positive association with lactose intake has been observed (Fairfield *et al*, 2004; Larsson *et al*, 2004). Most attention has been given to lactose even though other components of milk and milk products may be involved, such as dairy fat (Cramer *et al*, 1984; Qin *et al*, 2005), which may also be considered as a candidate risk factor because it is believed to exert an effect through hormone-related mechanisms.

To investigate whether consumption of dairy products (such as milk, yoghurt or cheese), its components (such as lactose or dairy fat) or its processing (fermentation) is associated with epithelial ovarian cancer risk, we used data from a large prospective cohort study conducted among postmenopausal women in the Netherlands, a country with high consumption of dairy products.

## MATERIALS AND METHODS

### The cohort

The Netherlands Cohort Study on Diet and Cancer is a prospective cohort study that started in September 1986 with the enrolment of 120 852 subjects (62 573 females) aged between 55 and 69 years from 204 municipalities with computerised population registries, located throughout the country. A detailed description of the study design has been published elsewhere (van den Brandt *et al*, 1990a). In brief, a case–cohort approach was used for data processing and analysis. Cases were enumerated from the entire cohort, whereas accumulated person-years for the entire cohort were estimated using a subcohort of 5000 participants, 2589 of whom were females. The subcohort was randomly sampled from the cohort after baseline exposure measurement and followed up biennially for vital status information. After 11.3 years of follow-up, information on vital status was available for all female subcohort members. After exclusion of women with prevalent cancer (other than skin cancer) at baseline and those who at baseline had reported to have undergone an oophorectomy, 2406 female subcohort members remained available for analyses.

### Identification of cases

Incident cancer cases were identified by computerised record linkage of the entire cohort to the regional cancer registries and to the nationwide network and registry of histo- and cytopathology in the Netherlands (PALGA) (van den Brandt *et al*, 1990a, 1990b). The completeness of cancer follow-up has previously been estimated to be over 95% (Goldbohm *et al*, 1994b). During 11.3 years of follow-up, 300 incident, microscopically confirmed, primary ovarian cancer cases (ICD-O-3:C56.9) were identified. After excluding nonepithelial tumours (*n*=9) and borderline invasive epithelial tumours (*n*=9), 282 invasive epithelial tumour cases remained available for analyses. Of these 282 epithelial ovarian cancers, 138 were serous carcinoma (ICD-O: 8260, 8441-8462, 9014).

### Questionnaire

Information on dietary habits and such potential confounders, as smoking behaviour, reproductive history, and family history of cancer, was collected at baseline, from all cohort members, using a self-administered questionnaire. This included a 150-item semiquantitative food frequency questionnaire, assessing regular food and beverage consumption in the year preceding the start of the study, which had been validated against a 9-day diet record, attaining Spearman's correlation coefficients of 0.60 for milk and milk products and 0.61 for cheese (Goldbohm *et al*, 1994a).

Data were obtained on frequency of dairy consumption (i.e., milk, yoghurt, cheese, curds, custard, oatmeal, butter, and pudding) and amount consumed on each consumption day (number of glasses for milk, number of slices of bread for cheese, and number of bowls for yoghurt, oatmeal, etc.). Consumption frequency was recorded using categories ranging from ‘never or less than once per month’ to ‘six or seven times per week’.

Sources of fermented milk and milk products were investigated, including yoghurt, buttermilk, fat cheese, and curds; for nonfermented milk and milk products these were milk and cream. Sources of lactose were yoghurt, milk, chocolate, butter, dry curd, custard, pudding, and oatmeal.

### Data analyses

After excluding subjects with incomplete or inconsistent dietary data (Goldbohm *et al*, 1994a), 252 ovarian cancer cases and 2216 subcohort members remained for analysis. Subjects were grouped into categories of dairy consumption (i.e., total milk, total yoghurt, and total cheese) and into quintiles of intake of lactose, dairy fat, fermented milk and milk products, and non-fermented milk and milk products, based on the distribution in the subcohort.

We examined if other risk factors (oral contraceptives use, (post)menopausal hormone therapy, parity, height, weight, body mass index, family history of ovarian or breast cancer, hysterectomy, age at menarche, age at menopause, tubal ligation, smoking behaviour, and socioeconomic status) were confounders in our data. Risk factors were considered to be confounders if they were associated with ovarian cancer risk, were associated with dairy intake, and, after inclusion in the model, changed the association of dairy with the risk of ovarian cancer by more than 10% compared to the model not including this factor. As a result, multivariable analyses included age (years), height (continuous), current cigarette smoker (y/n), duration of cigarette smoking (years), number of cigarettes smoked daily (continuous), duration of oral contraceptives use (years), and parity (continuous) as confounders.

Person-years of follow-up were calculated for the female subcohort members from the date they returned the questionnaire (September 1986) until the date of ovarian cancer diagnosis, death, or end of follow-up (December 1997).

Incidence rate ratios (RR) and corresponding 95% confidence intervals (CI) for ovarian cancer risk were estimated in age-adjusted and multivariate case–cohort analyses using Cox proportional hazards model (Cox, 1972) processed with the STATA statistical software package (Cleves *et al*, 2002). Standard errors were estimated using the robust Huber–White sandwich estimator to account for additional variance introduced by sampling from the cohort (Lin and Wei, 1989). The proportional hazards assumption was tested using the scaled Schoenfeld (1982) residuals. By fitting ordinal exposure variables as continuous terms, tests for dose-response trends in risks of ovarian cancer were carried out. Two-sided *P*-values are reported throughout the paper and considered statistically significant if <0.05.

## RESULTS

During 11.3 years of follow-up of 62 573 women, 282 epithelial ovarian cancer cases occurred, 252 of which were in women with complete and consistent dietary data. Of these 252 epithelial ovarian cancers, 126 were of the serous subtype.

Mean total dairy consumption as well as mean lactose intake were higher among women who reported ever use of oral contraceptives than among women who did not (Table 1), and was lower in current smokers than in nonsmokers. Taller women (>170 cm) also reported higher dairy consumption than those of lesser height (<160 cm). Lactose was highly correlated with total milk (*r*=0.73) intake, especially with respect to nonfermented milk and milk products (*r*=0.82). The correlation coefficient for dairy fat with total cheese was 0.46 and with total milk 0.27.

Table 2 shows the age-adjusted and multivariable-adjusted RR for ovarian cancer according to categories of milk, yoghurt, or cheese consumption. After adjusting for confounders, women in the highest category of total milk (RR=0.98, 95% CI=0.65–1.48), total yoghurt (RR=0.87, 95% CI=0.59–1.28), or total cheese (RR=1.06, 95% CI=0.54–2.08) consumption were not at greater risk of ovarian cancer compared to women in the lowest consumption category. Serous epithelial ovarian cancers did not show an association with the dairy products investigated.

No statistically significant association was found with consumption of fermented or nonfermented milk and milk products (Table 2). Only the highest quintile of dairy fat intake was associated with increased risk when compared to the lowest quintile of intake (RR_Q5 *vs* Q1_=1.53, 95% CI=1.00–2.36; *P*_trend_=0.11). This positive association was not seen for serous epithelial ovarian cancer (RR_Q5 *vs* Q1_=0.76, 95% CI=0.42–1.39). The numbers of other subtypes of epithelial ovarian cancer were too small to allow for separate analyses. The frequencies of all subtypes of epithelial ovarian cancer were uniformly distributed over the fat quintiles, with the exception of the endometrioid subtype (*n*=24), for which the frequency increased over the quintiles of dairy fat intake (data not shown).

Higher intake of lactose showed no association or at most a tendency towards decreased risk (for all ovarian cancers *P*_trend_=0.32 and for serous ovarian cancer *P*_trend_=0.11), largely due to a significantly decreased risk in the third quintile of intake for all epithelial ovarian cancers (RR_Q3 *vs* Q1_=0.62, 95% CI=0.39–0.98) as well as for serous ovarian cancers (RR_Q3 *vs* Q1_=0.45, 95% CI=0.22–0.91). The multivariable adjusted RR for lactose of the epithelial ovarian cancers or its serous subtype did not importantly change after additionally adjusting for dietary fat, energy, or total milk intake, respectively.

In addition, we investigated if the presence of subclinical disease at baseline had influenced our results, and re-analysed our data after excluding cases and subcohort members with less than 2 years of follow-up; this did not substantially change our results.

## DISCUSSION

In this large prospective cohort study of postmenopausal Dutch women, no relation between consumption of milk, yoghurt, or cheese and ovarian cancer risk was seen. In contrast to the previously reported results from cohort studies (Fairfield *et al*, 2004; Larsson *et al*, 2004), in the present study, lactose intake was not positively associated with serous ovarian cancer. The highest quintile of dairy fat intake was associated with increased ovarian cancer risk when compared to the lowest intake, but no statistically significant dose–response relation was observed and it was absent for serous ovarian cancers.

Contrary to our results, the Swedish (Larsson *et al*, 2004) and the US cohort studies (Kushi *et al*, 1999; Fairfield *et al*, 2004) have reported increased risk with higher consumption of milk. In the Swedish Mammography Cohort Study, the risk was 1.3 (95% CI=0.9–1.9) for women who consumed ⩾2 glasses milk per day compared to never or seldom users, and this association was stronger (RR=2.0, 95% CI=1.1–3.7) for serous ovarian cancer (Larsson *et al*, 2004). In the Nurses' Health Study, when skim or low-fat and whole milk were considered separately, a significant positive trend was only observed for skim or low-fat milk (Fairfield *et al*, 2004). Higher consumption of skim milk was also found to increase the risk of ovarian cancer in the Iowa Women's Health Study (Kushi *et al*, 1999). However, results from case–control studies have been inconsistent, some reporting no association with whole milk (Cramer *et al*, 2000; Goodman *et al*, 2002) or skim or low-fat milk (Cramer *et al*, 2000), whereas a positive association with whole milk and a negative association with skimmed milk have also been found (Mettlin and Piver, 1990; Webb *et al*, 1998). For consumption of yoghurt, a positive association with serous ovarian cancer was reported from the Nurses' Health Study, and for consumption of hard cheese the risk of all epithelial ovarian tumours was decreased with higher consumption (Fairfield *et al*, 2004). The results of our prospective cohort study are in accordance with results from previous case–control studies (Cramer *et al*, 2000; Goodman *et al*, 2002) in showing no effect of higher yoghurt or cheese consumption on ovarian cancer risk.

Lactose or dairy fat has been considered as the explanation for earlier findings of increased ovarian cancer risk with higher consumption of dairy products. In this regard, lactose and in particular its metabolite galactose have attracted most attention, since ovaries may be prone to galactose toxicity owing to their high local concentration of the galactose-metabolising enzyme galactose-1-phosphate uridyltransferase, as well as their high tissue-specific activity of this enzyme (Xu *et al*, 1989; Larsson *et al*, 2004). Galactose is believed to be ootoxic or to induce high concentrations of gonadotropins (Cramer, 1989; Cramer *et al*, 1989). Although in three prospective studies no statistically significant associations between all epithelial ovarian cancers and lactose intake has been reported (Kushi *et al*, 1999; Fairfield *et al*, 2004; Larsson *et al*, 2004), two of these studies did suggest that lactose might adversely affect serous ovarian cancer risk (Fairfield *et al*, 2004; Larsson *et al*, 2004). Our study results however suggest no association with lactose intake or might even indicate an inverse association. Inverse associations between lactose intake and ovarian cancer have previously been reported from case–control studies (Herrinton *et al*, 1995; Goodman *et al*, 2002). Differences in level of lactose intake may have contributed to the discrepancy among cohort studies, since the Netherlands has a high consumption of dairy products. However, categories of lactose intake did not differ largely among these studies although the median intake in the lowest categories was somewhat higher in the present Dutch than in the Swedish or US studies. The comparable number of ovarian cancer cases as well as of the serous subtype in the cohort studies would not explain the negative findings of the present study. Although height was found to be a risk factor in our cohort (Schouten *et al*, 2003), omitting height from the multivariate model did not change the RRs for total milk consumption or for lactose intake. Moreover, when included in the models, the interaction terms (height*lactose and height*total milk, respectively) did not reach statistical significance, thereby excluding height as an effect modifier. On the other hand, dairy fat intake was associated with risk in our study; women in the highest quintile of dairy fat intake were at 1.53 (95% CI=1.00–2.36) times higher risk than those in the lowest quintile. In 1990, Mettlin and Piver suggested that it was not the lactose content of milk but rather its fat content that was responsible for the association. High consumption of fat may influence ovarian cancer risk through increased oestrogen levels (Qin *et al*, 2005). Since this effect was absent for serous ovarian cancer, we hypothesise that dairy fat may present a risk for ovarian cancers other than serous, such as the endometrioid subtype, but their small number (*n*=24) did not allow for separate analysis.

Consumption of fermented dairy products was not associated with risk in our study. Fermented milk bacteria potentially have protective effects, because they provide a detoxification mechanism through the binding of heterocyclic aromatic amines directly to their cell walls (Knasmuller *et al*, 2001). Milk and other dairy products however also contain calcium and various vitamins, and moreover may be contaminated by pesticides; none of these were covered in the present study.

The important strengths of our study are its prospective design, reducing the potential for recall bias and the nearly complete follow-up of cases as well as of subcohort members, making selection bias unlikely. Moreover, we were able to control for confounding by most known ovarian cancer risk factors. Measurement error may have influenced our results, but we expect this to be random and to have at most biased our results towards the null. In addition, multivariate modelling itself may have added some uncertainty through measurement error affecting confounders as well (Schatzkin and Kipnis, 2004). We used a food frequency questionnaire to measure consumption of milk and milk products, which, in a validation study, was found to correlate well with a 9-day diet record (Goldbohm *et al*, 1994a). There was a relatively high correlation between lactose and total milk (*r*=0.73), but the risk estimate with lactose intake did not importantly change when milk consumption was included in the analysis. In addition, adjusting for dietary fat or total energy did not significantly change the RR for lactose.

In this prospective cohort study of postmenopausal women, consumption of milk, yoghurt or cheese was not associated with ovarian cancer risk. Contrary to previous findings, lactose intake was not associated with risk of serous ovarian cancer or even showed a slight inverse association. The highest quintile of dairy fat intake was associated with overall, but not with serous, ovarian cancer risk when compared to the lowest intake.

## Figures and Tables

**Table 1:** Mean (±s.d.) daily consumption of dairy products and lactose intake in the subcohort (*n*=2216), according to potential risk factors for ovarian cancer (recorded at baseline), Netherlands Cohort Study on Diet and Cancer, 1986–1997

		**Total dairy**	**Lactose**
**Characteristics**	***n* (%)**	**Mean (±s.d.)^a^**	**Mean (±s.d.)^a^**
*Age (years)*			
55–59	860 (38.8)	304.5 (196.1)	14.6 (8.5)
60–64	744 (33.6)	298.1 (179.6)	14.3 (7.8)
65–69	612 (27.6)	293.4 (188.2)	14.3 (8.3)

*Ever use of oral contraceptives*			
No	1643 (75.1)	293.4 (190.8)	14.2 (8.3)
Yes	545 (24.9)	319.6 (180.1)	15.3 (8.0)

*Ever use of (post)menopausal hormone therapy*			
No	1838 (86.9)	296.2 (188.1)	14.3 (8.2)
Yes	277 (13.1)	318.2 (189.3)	15.2 (8.2)

*Parity*			
0 children	398 (18.2)	298.9 (189.3)	14.2 (8.1)
1 child	179 (8.2)	296.2 (191.8)	14.6 (8.8)
2 children	472 (21.6)	301.3 (193.5)	14.2 (8.4)
>2 children	1136 (52.0)	298.7 (184.2)	14.5 (8.0)

*Hysterectomy*			
No	1838 (82.9)	296.4 (189.4)	14.3 (8.3)
Yes	378 (17.1)	313.5 (183.8)	15.0 (8.1)

*Family history of ovarian and/or breast cancer* b			
No	2023 (91.3)	301.0 (188.7)	14.5 (8.3)
Yes	193 (8.7)	281.2 (185.3)	13.5 (8.0)

*Tubal ligation*			
No	2206 (99.6)	299.2 (188.7)	14.4 (8.2)
Yes	10 (0.4)	310.7 (138.1)	14.5 (5.4)

*Age at menarche (years)*			
⩽12	569 (26.0)	293.3 (183.7)	14.0 (8.0)
13–14	995 (45.4)	303.3 (190.1)	14.6 (8.4)
⩾15	626 (28.6)	300.5 (188.4)	14.6 (8.1)

*Age at menopause (years)*			
⩽44	327 (15.8)	279.3 (182.2)	13.6 (8.0)
45–49	671 (32.5)	300.6 (185.8)	14.6 (8.1)
50–54	910 (44.0)	308.1 (193.3)	14.8 (8.5)
⩾55	158 (7.7)	291.2 (185.5)	14.2 (8.3)

*Current cigarette smoking*			
No	1746 (78.8)	304.3 (184.8)	14.6 (8.0)
Yes	470 (21.2)	280.5 (200.6)	13.8 (8.9)

*Socioeconomic status (highest level of education)*			
Primary school	741 (33.6)	301.2 (182.7)	14.7 (8.1)
Lower vocational school	511 (23.2)	284.4 (176.6)	13.9 (7.7)
High school/intermediate vocational school	760 (34.5)	301.3 (199.8)	14.3 (8.7)
Higher vocational school/University	191 (8.7)	322.2 (189.8)	15.1 (8.2)

*Height (in cm)*			
<160	510 (23.7)	272.2 (180.0)	13.2 (7.8)
160–164	403 (18.7)	282.9 (193.4)	13.7 (8.7)
164–167	428 (19.9)	314.2 (183.9)	15.1 (7.9)
167–170	441 (20.5)	299.8 (186.6)	14.6 (8.3)
>170	373 (17.3)	333.4 (193.0)	15.8 (8.4)

Due to missings, numbers do not always add up to the total.

a In g day^−1^.

b In first degree relatives (mother, sister, daughter).

**Table 2:** Age-adjusted and multivariate RRs and 95% CI for ovarian cancer according to dairy consumption and intake of fat or lactose, Netherlands Cohort Study on Diet and Cancer, 1986–1997

	**All invasive epithelial ovarian tumours**					**Serous epithelial ovarian tumours**			
	**Median**	**Cases/person years^a^**	**RR (95% CI)^a^**	**Cases/person years^b^**	**RR (95% CI)^b^**	**Cases/person years^a^**	**RR (95% CI)^a^**	**Cases/person years^b^**	**RR (95% CI)^b^**
*Total milk* (*g day*^−*1*^)									
0	0	86/8021	1.00	75/7347	1.00	44/8021	1.00	39/7347	1.00
0.1–<66.0	60	36/3677	0.91 (0.61–1.37)	33/3213	0.97 (0.63–1.50)	14/3677	0.69 (0.37–1.28)	12/3213	0.68 (0.35–1.32)
66.0–<150.1	119	33/3048	1.01 (0.66–1.55)	26/2733	0.95 (0.59–1.54)	21/3048	1.26 (0.74–2.16)	15/2733	1.04 (0.56–1.93)
150.1–<186.0	171	53/4778	1.02 (0.71–1.46)	49/4352	1.10 (0.75–1.62)	28/4778	1.04 (0.64–1.70)	26/4352	1.08 (0.64–1.81)
⩾186.0	343	44/4269	0.96 (0.66–1.41)	40/3849	0.98 (0.65–1.48)	19/4269	0.81 (0.47–1.41)	17/3849	0.79 (0.43–1.40)
*P*-trend			0.99		0.97		0.45		0.64

*Total yoghurt* (*g* *day*^−*1*^)									
0	0	69/6298	1.00	60/5677	1.00	37/6298	1.00	33/5677	1.00
0.1–<48.1	12	58/5507	1.00 (0.69–1.45)	54/4939	1.07 (0.72–1.59)	25/5507	0.80 (0.47–1.36)	22/4939	0.77 (0.44–1.35)
48.1–<96.2	53	58/4815	1.13 (0.78–1.64)	52/4473	1.11 (0.74–1.65)	34/4815	1.23 (0.76–1.99)	31/4473	1.16 (0.69–1.94)
⩾96.2	139	67/7174	0.88 (0.61–1.25)	57/6406	0.87 (0.59–1.28)	30/7174	0.73 (0.45–1.21)	23/6406	0.60 (0.34–1.05)
* P*-trend			0.61		0.63		0.19		0.1

*Total cheese* (*g* *day*^−*1*^)									
0	0	18/1754	1.00	15/1597	1.00	10/1754	1.00	9/1597	1.00
0.1–<12.9	7	61/5812	1.01 (0.58–1.76)	56/5160	1.21 (0.66–2.21)	28/5812	0.83 (0.40–1.75)	27/5160	0.93 (0.43–2.03)
12.9–<18.6	19	60/6254	0.92 (0.52–1.60)	53/5722	0.98 (0.53–1.81)	32/6254	0.88 (0.43–1.83)	26/5722	0.77 (0.35–1.69)
18.6–<37.1	31	82/6953	1.13 (0.66–1.94)	72/6297	1.20 (0.66–2.20)	39/6953	0.96 (0.47–1.97)	33/6297	0.86 (0.40–1.86)
⩾37.1	56	31/3022	1.01 (0.55–1.86)	27/2718	1.06 (0.54–2.08)	17/3022	0.99 (0.45–2.22)	14/2718	0.84 (0.35–2.03)
*P*-trend			0.85		0.8		0.97		0.96

*Fermented milk and milk products* (*g* *day*^−*1*^)									
0–<11	0	57/4759	1.00	52/4284	1.00	31/4759	1.00	28/4284	1.00
11–<53.4	28	49/4847	0.87 (0.58–1.30)	44/4375	0.81 (0.53–1.25)	20/4847	0.65 (0.36–1.15)	18/4375	0.62 (0.34–1.16)
53.4–<119.3	96	61/4745	1.10 (0.75–1.62)	52/4276	1.01 (0.67–1.53)	31/4745	1.02 (0.61–1.71)	25/4276	0.89 (0.51–1.57)
119.3–<192.7	144	43/4532	0.81 (0.53–1.23)	38/4195	0.72 (0.46–1.13)	22/4532	0.77 (0.44–1.34)	18/4195	0.63 (0.34–1.15)
⩾192.7	278	42/4911	0.74 (0.48–1.13)	37/4365	0.67 (0.42–1.06)	22/4911	0.71 (0.40–1.26)	20/4365	0.64 (0.35–1.18)
*P*-trend			0.31		0.26		0.40		0.37

*Nonfermented milk and milk products* (*g* *day*^−*1*^)									
0–<46	21	45/4736	1.00	40/4359	1.00	24/4736	1.00	22/4359	1.00
46–<115.8	77	49/4688	1.11 (0.72–1.69)	44/4158	1.16 (0.73–1.83)	22/4688	0.92 (0.51–1.67)	20/4158	0.93 (0.50–1.76)
115.8–<192.7	155	50/4810	1.09 (0.72–1.67)	42/4398	1.05 (0.66–1.67)	32/4810	1.31 (0.76–2.26)	25/4398	1.10 (0.60–2.02)
192.7–<292.0	230	61/4811	1.32 (0.88–1.98)	55/4337	1.32 (0.85–2.04)	27/4811	1.07 (0.61–1.89)	24/4337	1.01 (0.55–1.84)
⩾292.0	375	47/4750	1.05 (0.68–1.61)	42/4244	1.04 (0.65–1.65)	21/4750	0.87 (0.48–1.58)	18/4244	0.76 (0.40–1.47)
*P*-trend			0.71		0.70		0.63		0.85

*Dairy fat* (*g* *day*^−*1*^)									
0–<7.9	5	47/5191	1.00	43/4649	1.00	29/5191	1.00	28/4649	1.00
7.9–<12.7	10	49/5052	1.07 (0.70–1.63)	44/4731	0.98 (0.63–1.53)	26/5052	0.92 (0.53–1.58)	23/4731	0.75 (0.42–1.33)
12.7–<19.4	15	46/4776	1.07 (0.70–1.64)	40/4256	1.00 (0.63–1.59)	22/4776	0.82 (0.46–1.45)	18/4256	0.64 (0.35–1.19)
19.4–<31.0	24	46/4685	1.07 (0.70–1.64)	40/4231	0.92 (0.58–1.47)	24/4685	0.90 (0.52–1.56)	20/4231	0.67 (0.37–1.22)
⩾31.0	40	64/4091	1.70 (1.14–2.54)	56/3628	1.53 (1.00–2.36)	25/4091	1.04 (0.60–1.80)	20/3628	0.76 (0.42–1.39)
*P*-trend			0.04		0.11		0.94		0.63

*Lactose* (*g* *day*^−*1*^)									
0–<7.7	5	59/4759	1.00	52/4310	1.00	29/4759	1.00	25/4310	1.00
7.7–<11.7	10	49/4824	0.82 (0.55–1.22)	44/4393	0.87 (0.57–1.33)	31/4824	1.06 (0.63–1.79)	28/4393	1.11 (0.64–1.94)
11.7–<15.6	14	39/4755	0.66 (0.43–1.01)	33/4410	0.62 (0.39–0.98)	16/4755	0.55 (0.30–1.03)	12/4410	0.45 (0.22–0.91)
15.6–<21.1	18	57/5196	0.89 (0.61–1.31)	52/4620	0.92 (0.61–1.40)	29/5196	0.92 (0.54–1.57)	27/4620	0.95 (0.54–1.68)
⩾21.1	26	48/4260	0.92 (0.61–1.38)	42/3762	0.93 (0.60–1.45)	21/4260	0.81 (0.46–1.45)	17/3762	0.72 (0.37–1.38)
*P*-trend			0.41		0.32		0.29		0.11

a Age-adjusted analyses.

b Analyses adjusted for age (years), height (cm), current cigarette smoker (y/n), duration of cigarette smoking (years), number of cigarettes smoked daily, duration of oral contraceptive use (years) and parity (continuous), and fermented dairy products and nonfermented dairy products for each other.
